# Carcinogenic Role and Clinical Significance of Histone H3-H4 Chaperone Anti-silencing Function 1 B (ASF1B) in Lung Adenocarcinoma

**DOI:** 10.7150/jca.88777

**Published:** 2024-01-01

**Authors:** Congkuan Song, Yaolin Song, Xiaoxia Wan, Zhihong Zhao, Qing Geng

**Affiliations:** 1 Department of Thoracic Surgery, Renmin Hospital of Wuhan University, Wuhan, China.; 2 Department of Thoracic Surgery, Ezhou Central Hospital, Ezhou, China.

**Keywords:** Anti-silencing function 1B (ASF1B), lung adenocarcinoma (LUAD), prognosis, immunotherapy, biomarker.

## Abstract

Histone H3-H4 chaperone anti-silencing function 1 (ASF1) plays an important role in the polymerization, transport, and modification of histones. However, the significance of ASF1B in lung adenocarcinoma (LUAD) is largely overlooked. We investigated the aberrant expression of ASF1B in LUAD and its potential link to patient survival using multiple databases. ASF1B-overexpressing and knockdown cell lines were constructed to explore its effects on the biological behavior of lung cancer cells. ssGSEA, TMB, TIDE and IMvigor210 cohort were used to explore and validate the association of ASF1B to tumor immunity. Our data suggested that ASF1B was overexpressed in LUAD, and was associated with poor prognosis. ASF1B promoted the proliferation, migration, and invasion of lung cancer cells by regulating the phosphorylation of AKT *in vitro*. ASF1B was associated with tumor immunity. In summary, ASF1B may promote malignant behavior of LUAD cells, and its overexpression correlates with worse prognosis and better immunotherapy effect.

## Introduction

Lung cancer is one of the malignant tumors with high morbidity and mortality, with complex and diverse etiology 1. Lung adenocarcinoma (LUAD) is the most common form of non-small cell lung cancer (NSCLC), accounting for approximately 40% of lung cancer cases 2. Clinically, patients with LUAD often lack typical clinical symptoms or even have no symptoms in the early stage, and LUAD are prone to distant metastasis and high drug resistance. These characteristics also make the clinical treatment of LUAD extremely challenging. The treatment of lung cancer is mainly surgery, radiotherapy and chemotherapy. In recent years, with the rapid development of medicine, the treatment methods of lung cancer are gradually enriched, and gene targeted therapy and immunotherapy are also gradually emerging. Despite the importance of these new therapies for improving patient outcomes, some patients with LUAD still lack responses to these or inevitably develop drug resistance with poor prognosis. Therefore, further studies are still necessary to better understand the mechanisms underlying the development of LUAD in order to explore more alternative treatment strategies.

The anti-silencing function 1 (ASF1), as the histone H3-H4 chaperone, was initially identified in yeast 3. It plays an important role in histone polymerization, transport, modification and other processes, as well as in DNA replication, DNA damage repair and transcriptional regulation 4-7. Previous studies 8, 9 have shown that ASF1B is dysregulated in multiple malignancies and can regulate cell proliferation, apoptosis and cell cycle. However, its expression pattern, molecular mechanisms and clinical significance in LUAD are not fully understood. Here, we performed a systematic analysis of ASF1B using multiple databases, explored the expression of ASF1B in different cancers and its association with prognosis, and highlighted the importance of ASF1B in LUAD. Moreover, we induced the downregulation and overexpression of ASF1B in LUAD cell lines, examined its effects on the biological behavior of lung cancer cells *in vitro*, and preliminarily revealed the molecular mechanism that ASF1B can promote malignant behavior of LUAD cells by affecting the phosphorylation of AKT. Finally, we also explored the potential associations between ASF1B expression levels and tumor immune infiltration as well as the efficacy of immunotherapy. Taken all together, our data further confirm the importance of ASF1B in LUAD and provide new insights into the role of ASF1B in the prognosis and immunity in LUAD.

## Materials and Methods

### Data Acquisition

From the Cancer Genome Atlas (TCGA) database (https://cancergenome.nih.gov), we downloaded LUAD data, including the transcriptional profile data of 535 tumor tissues and 35 normal lung tissues and the corresponding clinical data. The LUAD gene expression profiles were also downloaded from the Gene Expression Omnibus (GEO) (https://www.ncbi.nlm.nih.gov/). ASF1B differentially expressed in lung tumor tissues versus normal tissues was examined using the follow three datasets: TCGA-LUAD, GSE10072 10 and GSE32863 11. In order to determine the impact of ASF1B on LUAD prognosis, these datasets including TCGA-LUAD, GSE30219 12, GSE31210 13, 14, GSE42127 15, 16, GSE68465 17, and GSE72094 18 were employed. We obtained tumor and adjacent tissue specimens from 12 LUAD patients admitted to the Renmin Hospital of Wuhan University in June 2023. Acquisition of these specimens was approved by the Ethical Review Board of Renmin Hospital of Wuhan University (Approval No.: WDRY2023-K112). Furthermore, protein expression difference of ASF1B between lung tumor tissues and normal tissues was validated using the Human Protein Atlas (HPA) database (http://www.proteinatlas.org/) and UALCAN (http://ualcan.path.uab.edu/). In addition, the subcellular proteome of ASF1B was also investigated in the HPA database.

### Pan-cancer analysis

ASF1B expression in pan-cancers was explored in TIMER2 database (http://timer.cistrome.org/). The effect of ASF1B on the prognosis of pan-cancer was analyzed in GEPIA2 webserver (http://gepia2.cancer-pku.cn/). The correlation of ASF1B with the immune checkpoint was explored in TISIDB database (http://cis.hku.hk/TISIDB/).

### ssGSEA analysis for immune cells and immune pathways

Single-sample gene set enrichment analysis (ssGSEA) was conducted to to quantify the score of 16 immune cells and 13 immune-related pathways or functions. Wilcoxon test was conducted to compare the immune cell and immune function scores between high and low ASF1B expression groups. The relationship between ASF1B and these scores was also examined using Spearman correlation analysis by Xiantao webserver (https://www.xiantaozi.com/).

### Cell culture

LUAD A549 cells and human normal epithelial cells of lung (Beas-2B) were obtained from ATCC. Fetal bovine serum (FBS) were purchased from Gibco, USA. And penicillin/streptomycin and RPMI 1640 medium were from Servicebio, Wuhan. A549 cells were cultured using RPMI 1640 medium containing 10% FBS, 50 mg/mL streptomycin and 50 IU/mL penicillin, and placed in an incubator conditioned at 37℃, 5% CO_2_. The solution was changed every 1~2 d, and when the cell fusion reached 80%~90%, it was digested with trypsin and passaged at 1:3.

### Construction of the ASF1B-overexpressing and knockdown cell lines

According to the human ASF1B gene sequence published by GenBank, we used an online software (https://www.ncbi.nlm.nih.gov/tools/primer-blast/) to design the corresponding primers, the target gene fragments were amplified by nested PCR, and the vector (pHAGE-3xflag) was then digested and purified. The recombinant vector was obtained by connecting the target gene fragment with the vector. They were subsequently transferred into E. coli. We screened the positive clones for expanded culture. PEI transfection reagent (sigma, # GF95977287), 0.5μg pMD2.G (addgene, plasmid #12259 pMD2.G), and 0.75μg psPAX2 (addgene, plasmid #12260 psPAX2) were thoroughly mixed, and then mixed with 1μg target gene plasmid or control vector plasmid respectively for 15 minutes, and then added to 293T cell culture medium for lentiviral packaging. The sequences of the short hairpin RNA against human ASF1B (ASF1B-sh#1, and ASF1B-sh#2) were respectively 5′-CTGGAGTGGAAGATCATTTAT-3′ and 5′-TTAGTTAGTAGGTAGACTTAG-3′. After 6 hours of packaging the virus, the liquid in the medium was changed. After 48 hours, the virus was collected and centrifuged for 3 minutes at 12000 rpm in a centrifuge, and then collected using filter membrane with a 0.45 μm pore size. The virus collected above was added to the A549 cell culture dish to infect the target cells, and 8 μg/mL polybrene (Sigma, H9268) was added to the two cell culture dishes respectively. After the cells were passaged, 4μg/mL puromycin reagent was used for screening the stably transduced cells, and the screening time was 48h.

### CCK-8 Assay

The cells were plated in 96-well plates (5*10^3^/well). We added 10 μl of CCK-8 (Servicebio, Wuhan) solution to the cell culture at each time point, and the cells were incubated for 2-4h at 37 ℃ and 5% CO_2_. We used a microplate reader to detect the absorbance value (OD value) in each well at 450 nm.

### EdU Assay

The stable ASF1B-overexpressing or knockdown cell lines with good growth status were seeded in 48-well plates with 10000 cells per well and cultured in an incubator at 37℃ and 5% CO_2_. When cells had been incubated with EdU (EdU kit were purchased from Servicebio, Wuhan) for 2 hours, the culture medium was removed, and paraformaldehyde was added to fix it at room temperature for 15 min. We washed the cells thrice with PBS. Next, we added 0.3% Triton X-100 permeable solution into each well. The cells were incubated at room temperature for 15 min and then washed with PBS for 3 times. After this, we added click reaction solution to each well. And the cells were incubated at 37℃ for 30 min away from light. We subsequently aspirated the click reaction solution, and washed the cells thrice with PBS. Nuclei staining was performed using Hoechst 33342.

### Transwell assay

Cell migration and invasion were measured by transwell assay. Matrigel glue, and Transwell culture plates were purchased from BD, USA. Cultured cells with good growth status were harvested and made single cell suspension after tryptic digestion. We added 100 µL of cell suspension to the transwell chamber for 14 h. Subsequently, we washed the chamber twice with calcium-free PBS. After paraformaldehyde fixation and crystal violet staining, we randomly selected five fields of view under a 100-fold or 200-fold microscope to observe the cells, and the number of migrated cells was counted. For the invasion assay, matrigel was added early in the upper chamber of a transwell chamber and incubated in the cell incubator for about 2 h. In the upper compartments of the transwell chamber, 100 µL of cell suspension was inoculated. And the lower compartments of the transwell chamber were filled with 600 µL of RPMI 1640 medium containing 20% FBS. The cells in the upper compartment were incubated at 37 °C for 24 h, then wiped with a wet cotton swab, fixed with 4% paraformaldehyde for 10 minutes, and stained with 0.5% crystal violet for 5 minutes. In order to observe if cells entered the bottom cavity via the pore, a microscope (Olympus, Japan) was used. And these images were taken and the number of cells was counted as well.

### Quantitative real-time polymerase chain reaction (qRT-PCR)

ASF1B mRNA expression in cells was measured using qRT-PCR analysis. Trizol reagents, qRT-PCR kit, and reverse transcription kit were purchased from Servicebio, Wuhan. Applying the Trizol method, the total RNA was extracted and subsequently used to synthesize cDNA and subjected to PCR reactions (all experimental procedures were performed strictly according to the instructions of the kit). β-ACTIN was used as the reference gene and relative gene expression was calculated by the 2^-ΔΔCT^ method.

### Western blot

In this experiment, we lysed cells for 30 minutes, centrifuged them, and absorbed the supernatant to extract total cellular protein. And the protein was quantitated using the BCA protein assay kit. Protein signals were detected by a BioRad ChemiDoc XRS+System. Relative protein expression levels were quantitated using β-actin as the internal reference. The relevant antibodies (ASF1B, AKT, p-AKT, β-actin) and MK2206 (#SF2712, an AKT-specific inhibitor) were purchased from Servicebio, Wuhan.

### Evaluation of the immunotherapy efficacy

There is a close relationship between immunotherapy effect and immune checkpoint expression, as well as tumor mutation burden (TMB), thus we also investigated the Spearman correlation between ASF1B and some immune checkpoints in GEPIA2 webserver, and compared the differences in TMB between high and low ASF1B expression groups. TCGA somatic mutation data were also collected from the TCGA GDC (https://portal.gdc.cancer.gov/). Additionally, due to tumour immune dysfunction and exclusion (TIDE) integrates the characteristics of T cell dysfunction and exclusion, and simulates the tumor immune escape with different levels of tumor infiltrating cytotoxic T cells, and has very prominent advantages over other biomarkers 19, we thus utilized the TIDE score from TIDE database (http://tide.dfci.harvard.edu) to predict the potential immunotherapy response. Furthermore, we obtained an immunotherapy dataset (IMvigor210) with a large sample size from a previous study 20 to further clarify the association between ASF1B expression and the immunotherapy effect.

### Statistical Analysis

All statistical analyses were performed in R 3.6.2 software, Graphpad Pism9.5, IBM SPSS Statistics 23.0 and online databases. Spearman method was used for correlation analysis. The median of ASF1B expression values for all samples was used as the cut-off for grouping (high and low ASF1B expression groups). “*” or “**^#^**” in Figures represents p-value less than 0.05, “**” or “**^##^**” represents p-value less than 0.01, “***” or “**^###^**” represents p-value less than 0.001. Kaplan-Meier curves were used to assess survival outcomes, and log-rank test was performed. P-value < 0.05 was considered statistically significant.

## Results

### Expression and relationship with prognosis of ASF1B in human cancer

**Figure 1:**
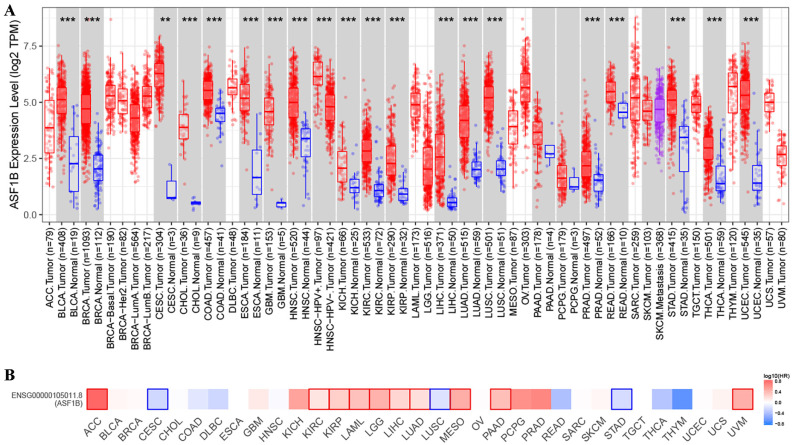
** ASF1B expression in pan-cancer and its relationship with prognosis. (A)** TIMER2 analysis revealed ASF1B expression in human pan-cancer. “*” in Figure represents p-value less than 0.05, “**” represents p-value less than 0.01, “***” represents p-value less than 0.001. **(B)** GEPIA2 analysis revealed the relationship between the expression of ASF1B and the patient outcome.

In TIMER2 analysis, we observed that ASF1B expression was dysregulated in multiple human cancers, including urinary system malignancies (bladder urothelial carcinoma (BLCA), kidney chromophobe (KICH), kidney renal clear cell carcinoma (KIRC), and kidney renal papillary cell carcinoma (KIRP)), digestive system malignancies (cholangiocarcinoma (CHOL), liver hepatocellular carcinoma (LIHC), pancreatic adenocarcinoma (PRAD), esophageal carcinoma (ESCA), stomach adenocarcinoma (STAD), colon adenocarcinoma (COAD), and rectum adenocarcinoma (READ)), nervous system malignancies (glioblastoma multiforme (GBM), brain lower grade glioma (LGG)), respiratory system malignancies (LUAD, lung squamous cell carcinoma (LUSC)), female reproductive system malignancies (cervical squamous cell carcinoma and endocervical adenocarcinoma (CESC), uterine corpus endometrial carcinoma (UCEC)), and breast invasive carcinoma (BRCA), head and neck squamous cell carcinoma (HNSC), and thyroid carcinoma (THCA). Surprisingly, in all of these 20 human cancers, ASF1B was highly expressed (**Figure 1A**). Subsequently, we investigated the effect of ASF1B expression on cancer prognosis. In GEPIA2 analysis, we found that ASF1B expression could significantly affect the survival of 13 cancers. For example, ASF1B overexpression was associated with worse prognosis in some cancers, including Adrenocortical carcinoma (ACC), KIRP, KIRC, and LUAD. Instead, ASF1B overexpression was associated with better prognosis in CESC, LUSC, and STAD (**Figure 1B and Figure 1S**). Overall, ASF1B is upregulated in a variety of human cancers, and is closely related to their prognosis.

### ASF1B is highly expressed in LUAD and correlates with poor prognosis

**Figure 2:**
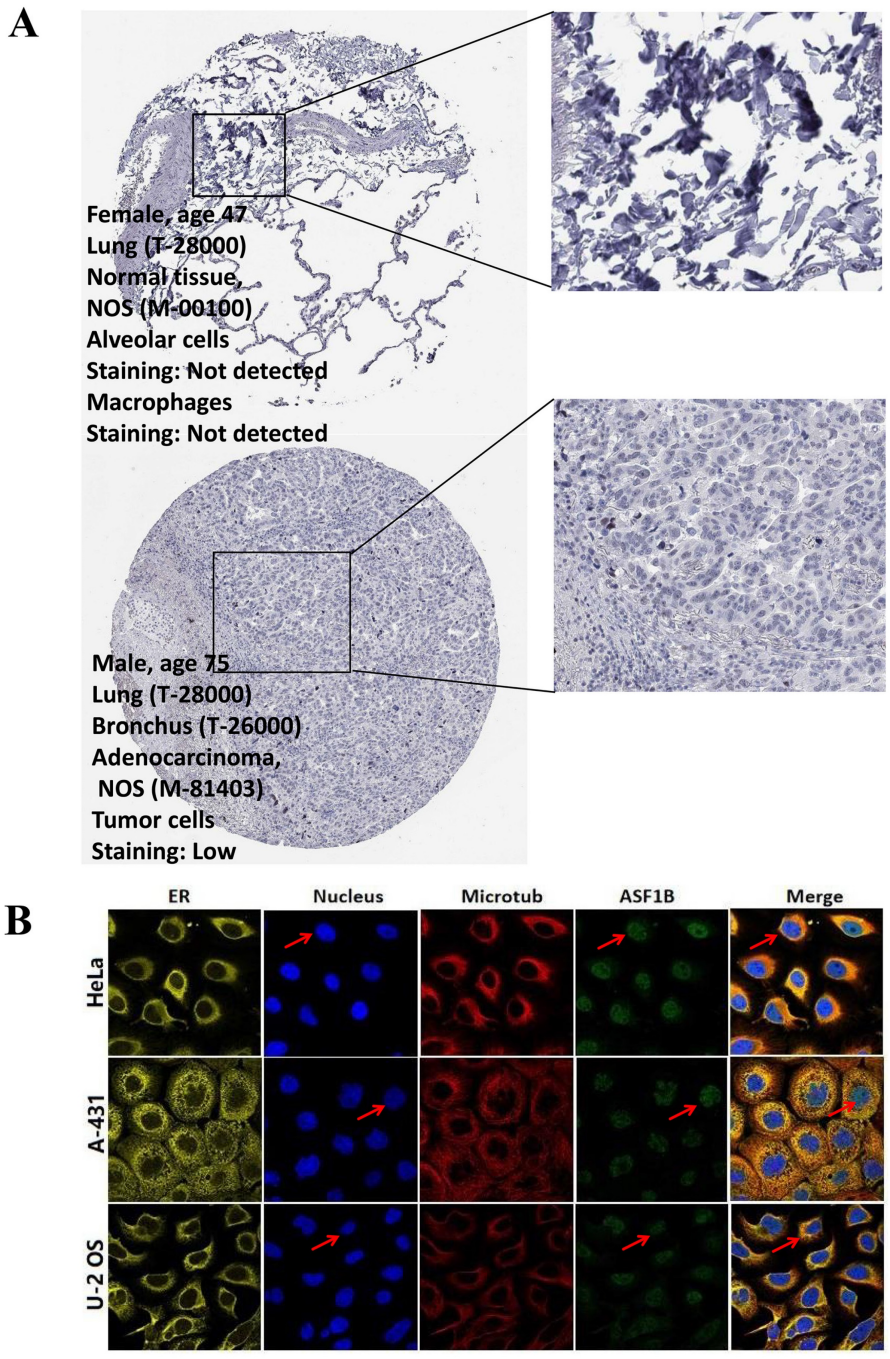
** The immunohistochemical results and subcellular proteome of ASF1B. (A)** The immunohistochemical results of ASF1B in LUAD tumor and normal tissues form HPA. **(B)** HPA database analysis revealed the subcellular proteome of ASF1B in human cancer cells (HeLa, A-431, and U-2 OS cells).

**Figure 3:**
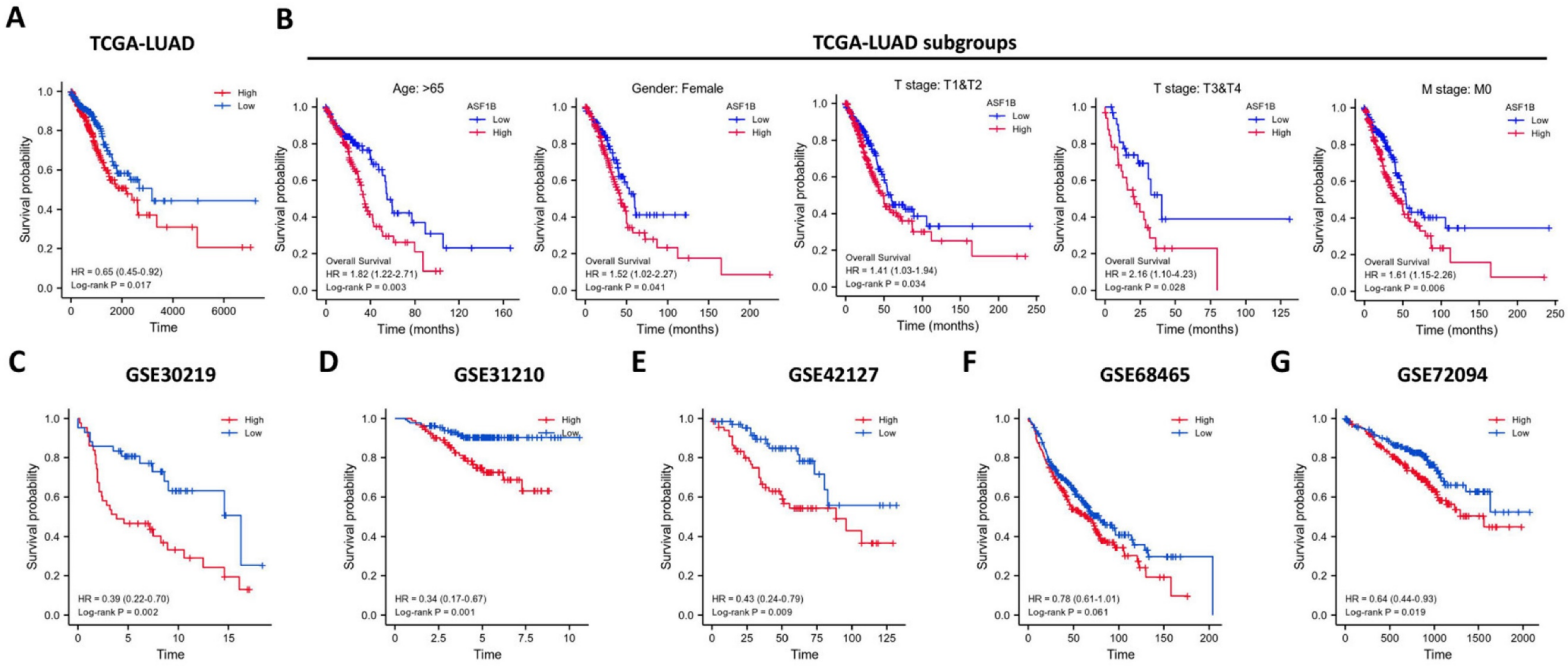
** Effect of ASF1B expression on patient overall survival. (A)** Kaplan-Meier curve reveals the relationship between ASF1B and the overall survival of all patients from TCGA-LUAD. (**B**) Kaplan-Meier survival curves based on TCGA-LUAD subgroups for age greater than 65 years, female patients, T1/T2, T3/T4, and stage M0. **(C-G)** Kaplan-Meier curve reveals the relationship between ASF1B and the overall survival of LUAD patients in the GEO datasets: GSE30219 (**C**), GSE31210 (**D**), GSE42127 (**E**), GSE68465 (**F**) and GSE72094 (**G**).

In the pan-cancer analysis described above, we initially observed the overexpressed status of ASF1B in LUAD and it was related to poor prognosis. To further clarify this relationship, we performed the validation in multiple datasets. In the TCGA-LUAD project, we first compared ASF1B expression between the two groups in unpaired LUAD tumor (n=535) and normal tissue samples (n=59), and found that compared to normal tissues, tumor tissues expressed significantly more ASF1B. In the 57 paired LUAD tumor and normal tissue samples, as expected, ASF1B remained highly expressed in the tumor tissue (**Figure S2A**). Similar results were seen in other data cohorts before and after the paired samples, including GSE10072 (**Figure S2B**) and GSE32863 (**Figure S2C**). At the cellular level, we also found that ASF1B expression was also significantly higher in LUAD cells compared to lung normal cells (**Figure S2D**). Similarly, ASF1B also showed significant expression differences in 12 pairs of LUAD tumors and adjacent normal tissues (**Figure S2E**). We also explored ASF1B expression at the protein level. In HPA analysis, we found that the expression level of ASF1B in tumors was higher than that in normal tissues (**Figure 2A**). From the results of immunohistochemistry, ASF1B is mainly expressed in the nucleus (**Figure 2A**). We also observed the same phenomenon (localized to the nucleoplasm) in human cancer cells (HeLa, A-431, and U-2 OS cells) in the HPA database (**Figure 2B**). Furthermore, we also observed protein expression level of ASF1B in tumors was higher than that in normal tissues (CPTAC samples) in UALCAN portal (**Figure S2F**). Similarly, we also verified the relationship between ASF1B and LUAD prognosis in multiple datasets. First, in TCGA-LUAD program, we found that the ASF1B overexpression was correlated with worse prognoses (**Figure 3A**), which was also confirmed in the subsequent TCGA subgroup analysis in multiple subsets, including age > 65y group, female group, stage T1&T2 group, stage T3&T4 group, and stage M0 group (**Figure 3B**). Similar results were seen in other LUAD cohorts, including GSE30219 (**Figure 3C**), GSE31210 (**Figure 3D**), GSE42127 (**Figure 3E**), GSE68465 (**Figure 3F**), and GSE72094 (**Figure 3G**). Moreover, to further investigate the association between ASF1B and clinical features, we found significant correlations between ASF1B expression and patient age, T stage, N stage, and TNM stage (**Table 1**). Together, these data confirm that ASF1B is overexpressed in LUAD and correlates with poor prognosis.

### ASF1B promotes the proliferation, migration, and invasion of lung cancer cells

**Figure 4:**
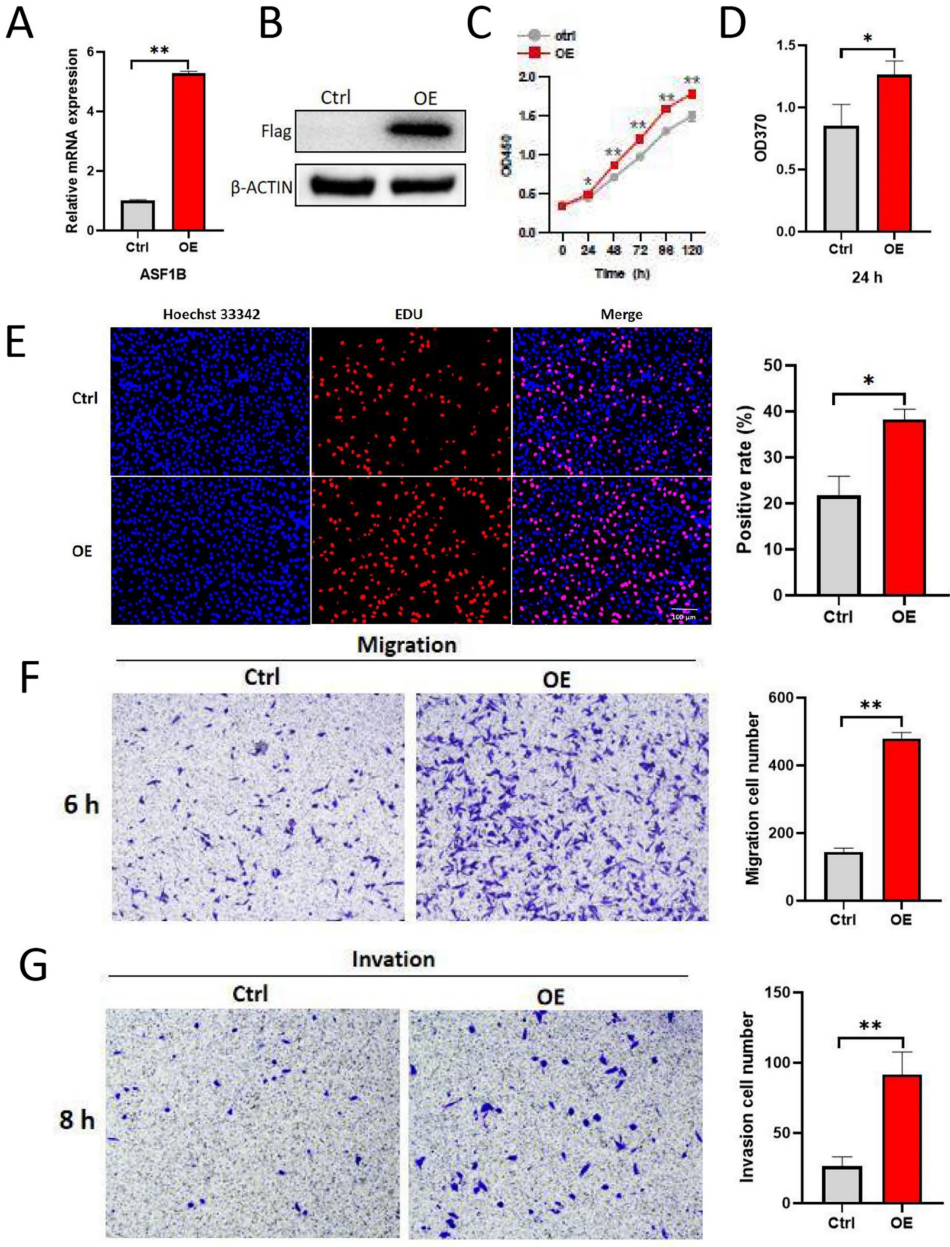
** ASF1B overexpression promotes the proliferation, migration and invasion of lung cancer cells. (A)** qRT-PCR revealed the mRNA expression levels of ASF1B in overexpressed cell lines versus control groups. **(B)** Western blot revealed the protein expression levels of ASF1B in overexpressed cell lines versus control groups. **(C)** CCK-8 assay showed the growth rate of ASF1B-overexpressing cells increased significantly with increasing time points. **(D)** BrdU assay revealed the OD value at 370 nm between the ASF1B overexpression and the control group cells at 24 h. **(E)** EdU assay revealed the cell proliferation in ASF1B overexpressed cell lines versus control groups. **(F)** Transwell assay revealed the migratory abilities of A549 cells. **(G)** Transwell assay revealed the invasive abilities of A549 cells (*p<0.05, **p<0.01, ***p<0.001).

To explore the impact of ASF1B on the biological behavior of lung cancer cells, ASF1B was overexpressed to construct stably expressed cell lines in A549. As shown in **Figure 4A-B**, ASF1B in this cell line was significantly overexpressed at the mRNA and protein levels. The result from CCK-8 assay (**Figure 4C**) showed the growth rate of ASF1B-overexpressing cell lines increased significantly with increasing time points, and BrdU assay (**Figure 4D**) also showed that the proliferative capacity of the A549 cell lines with ASF1B overexpression were significantly enhanced. These data suggested that ASF1B may act as an oncogene to promote lung cancer cell growth. Furthermore, to further confirm this finding, we also assessed the effect of ASF1B overexpression on lung cancer cell proliferation by EdU marker staining. As shown in **Figure 4E**, compared to the cells in the control group, ASF1B-overexpressing cells had a significantly higher positive rate for EdU staining, indicating that ASF1B overexpression can promote the proliferation of lung cancer cells. Since lung cancer is predisposed to invasion and metastasis, we used the Transwell assay to evaluate the effect of ASF1B on the migratory and invasive ability of lung cancer cells. ASF1B overexpressing cells significantly promoted the ability of tumor cells to penetrate the transwell compartment when compared with controls (**Figure 4F**). In addition, in the invasive cell experimental model, the overexpression of ASF1B also aggravated the cell invasive ability (**Figure 4G**).

**Figure 5:**
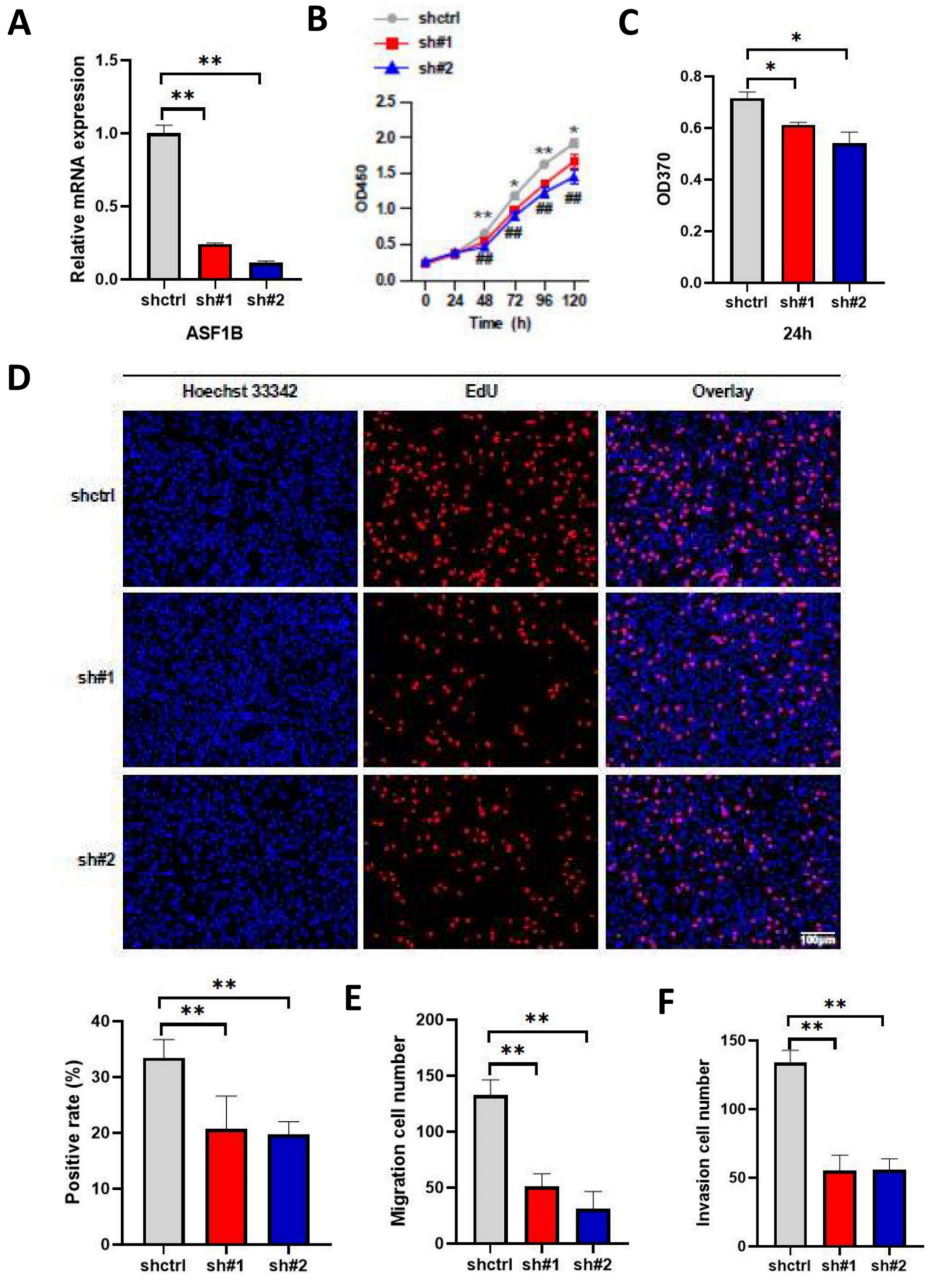
** ASF1B knockdown inhibits the proliferation, migration, and invasion of lung cancer cells**. (**A**) qRT-PCR revealed the mRNA expression levels of ASF1B in knockdown cell lines versus control groups. (**B**) CCK-8 assay showed the growth rate of ASF1B-knockdown cells decreased significantly with increasing time points (shctrl Vs. sh#1: *p<0.05, **p<0.01, ***p<0.001; shctrl Vs. sh#2: **^#^**p<0.05, **^##^**p<0.01, **^###^**p<0.001). (**C**) BrdU assay revealed the OD value at 370 nm between the ASF1B knockdown and the control group cells at 24 h. (**D**) EdU assay revealed the cell proliferation in ASF1B-knockdown cell lines versus control groups. (**E**) Transwell assay revealed the migratory abilities of A549 cells. (**F**) Transwell assay revealed the invasive abilities of A549 cells (*p<0.05, **p<0.01, ***p<0.001).

The above results suggested the effect of ASF1B overexpression on the proliferation, migration, and invasion ability of lung cancer cells. To further emphasize the impact of ASF1B on the biological behavior of lung cancer cells, we constructed ASF1B-knockdown cell lines by shRNA-mediated gene silencing in A549 cell lines. This result revealed that the ASF1B expression levels were significantly decreased in the ASF1B knockdown cell lines as compared to the ASF1B non-knockdown cell lines (**Figure 5A**), suggesting the transfection was successful. The CCK-8 assay (**Figure 5B**) and BrdU assay (**Figure 5C**) showed a significant decrease in cell growth rate and proliferation capacity in the ASF1B-sh#1 and ASF1B-sh#2 cell lines. EdU marker staining (**Figure 5D**) indicated that ASF1B deletion significantly decreased the proliferation of A549 cells. These results indicated that loss of ASF1B significantly inhibited the proliferative capacity of lung cancer cells. Meanwhile, the Transwell assay was also performed to explore the effect of ASF1B deletion on cell migration and invasion. We observed a significant decrease in the number of cells that migrated in the ASF1B knockdown group (ASF1B-sh#1 and ASF1B-sh#2) (**Figure 5E-F and Figure S3**).

Overall, the above data revealed that ASF1B had a major part to play in tumor cell proliferation, migration, and invasion. And targeting ASF1B may be a new and promising treatment strategy for LUAD.

### ASF1B promotes malignant behavior of LUAD cells by regulating the phosphorylation of AKT

**Figure 6:**
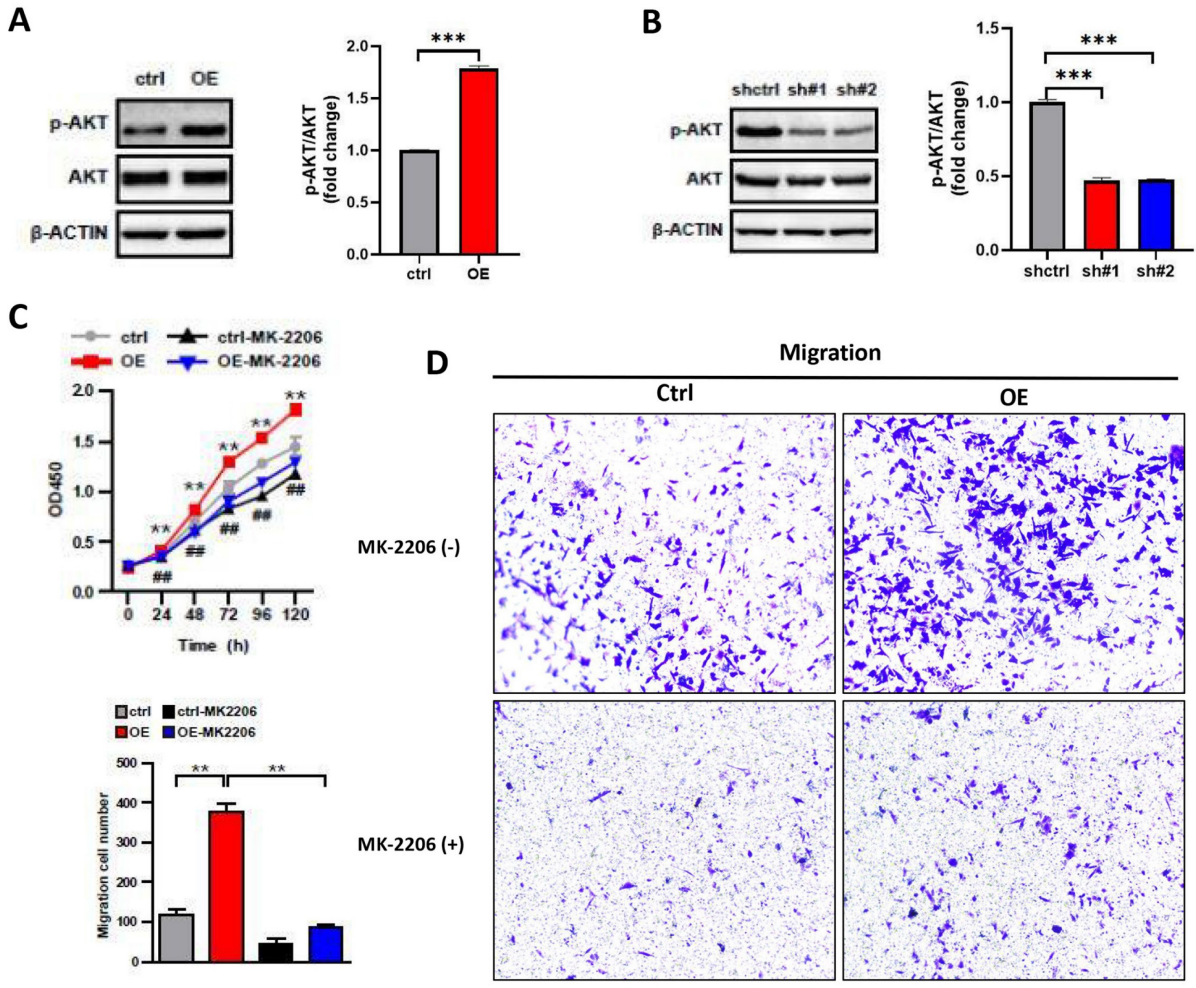
**ASF1B promotes malignant behavior of LUAD cells by regulating the phosphorylation of AKT. (A)** Western blot revealed the protein expression levels of AKT, p-AKT, β-actin in ASF1B overexpressed cell lines versus control groups. **(B)** Western blot revealed the protein expression levels of AKT, p-AKT, β-actin in ASF1B-knockdown cell lines versus control groups. **(C)** Cell activity of A549 cells in each group detected by CCK-8 (ctrl Vs. OE: *p<0.05, **p<0.01, ***p<0.001; ctrl Vs. Ctrl-MK-2206: **^#^**p<0.05, **^##^**p<0.01, **^###^**p<0.001). **(D)** Migratory abilities of A549 cells in each group detected by Transwell (*p<0.05, **p<0.01, ***p<0.001).

The PI3K/AKT pathway plays a key role in carcinogenesis, promoting cell survival and growth 21, and AKT is as the core to the PI3K/AKT pathway. To investigate whether the impact of ASF1B on the biological behavior of A549 cells was associated with AKT, we performed the following analysis. We examined the protein expression levels of AKT and phosphorylation-modified AKT (p-AKT) in lung cancer cells by Western blot. We observed that the overexpression of ASF1B significantly increased the phosphorylation level of AKT (**Figure 6A**), while the knockdown of ASF1B observably inhibited the AKT phosphorylation (**Figure 6B**). More importantly, MK2206 (an AKT-specific inhibitor), significantly inhibited the effects of ASF1B-overexpressing cell lines on tumor cell proliferation (**Figure 6C**) and migration (**Figure 6D**) *in vitro*, suggesting that AKT played a pivotal role in biological function of ASF1B in promoting lung cancer progression, and ASF1B promoted malignant behavior of LUAD cells by regulating the phosphorylation of AKT.

### Correlation of ASF1B expression with immune characteristics

**Figure 7:**
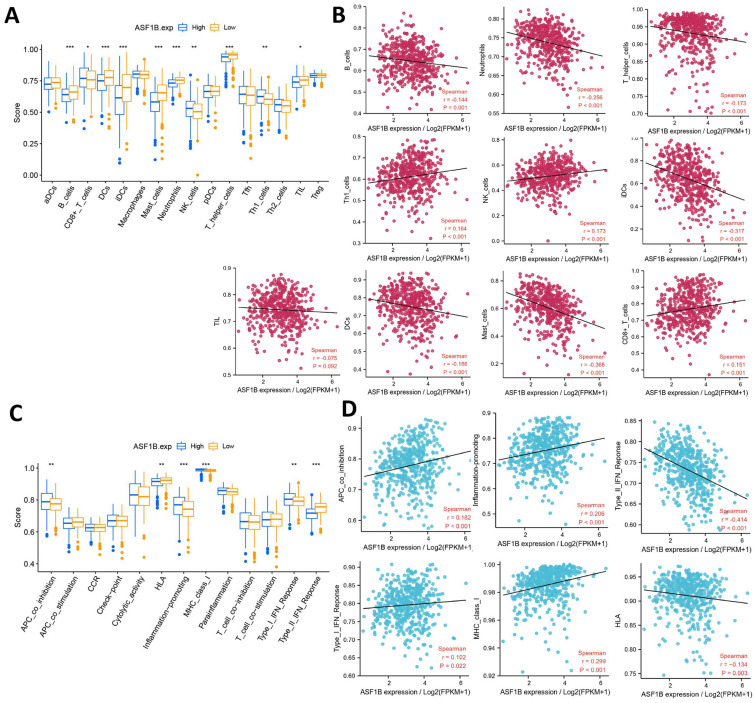
** Correlation of ASF1B expression with immune characteristics. (A)** Comparison of the abundance of immune cell infiltration between high and low ASF1B expression groups. **(B)** The relationship between ASF1B and immune cells examined by Spearman correlation analysis. **(C)** Comparison of the activity of immune-related pathways or functions between high and low ASF1B expression groups. **(D)** Spreaiman correlation between ASF1B expression and the activity of immune-related pathways or functions (*p<0.05, **p<0.01, ***p<0.001).

The relationship between ASF1B and cancer immunity is largely unknown. We thus investigated the immune characteristics of different expression groups of ASF1B using ssGSEA. Among the 16 immune cells, seven had higher infiltration abundance in the low ASF1B group, including B cells, DCs, iDCs, Mast cells, Neutrophils, TIL, T helper cells. While CD8^+^ T cells, NK cells, and Th1 cells had higher levels of infiltration in the high ASF1B group (**Figure 7A**). The association of these immune-infiltrating cells was also further confirmed by Spearman correlation analysis with ASF1B (**Figure 7B**). In addition, among the 13 immune-related pathways, four had lower activity in the low ASF1B group, including MHC class I, APC co-inhibition, Type I IFN Reponse, Inflammation promoting. While HLA, Type II IFN Reponse diaplayed higher activity in the low ASF1B group (**Figure 7C**). Similar results were also seen in the correlation analysis of ASF1B with the activity of these immune pathways (**Figure 7D**).

### ASF1B overexpression may indicate a better immunotherapy effect in LUAD patients

**Figure 8:**
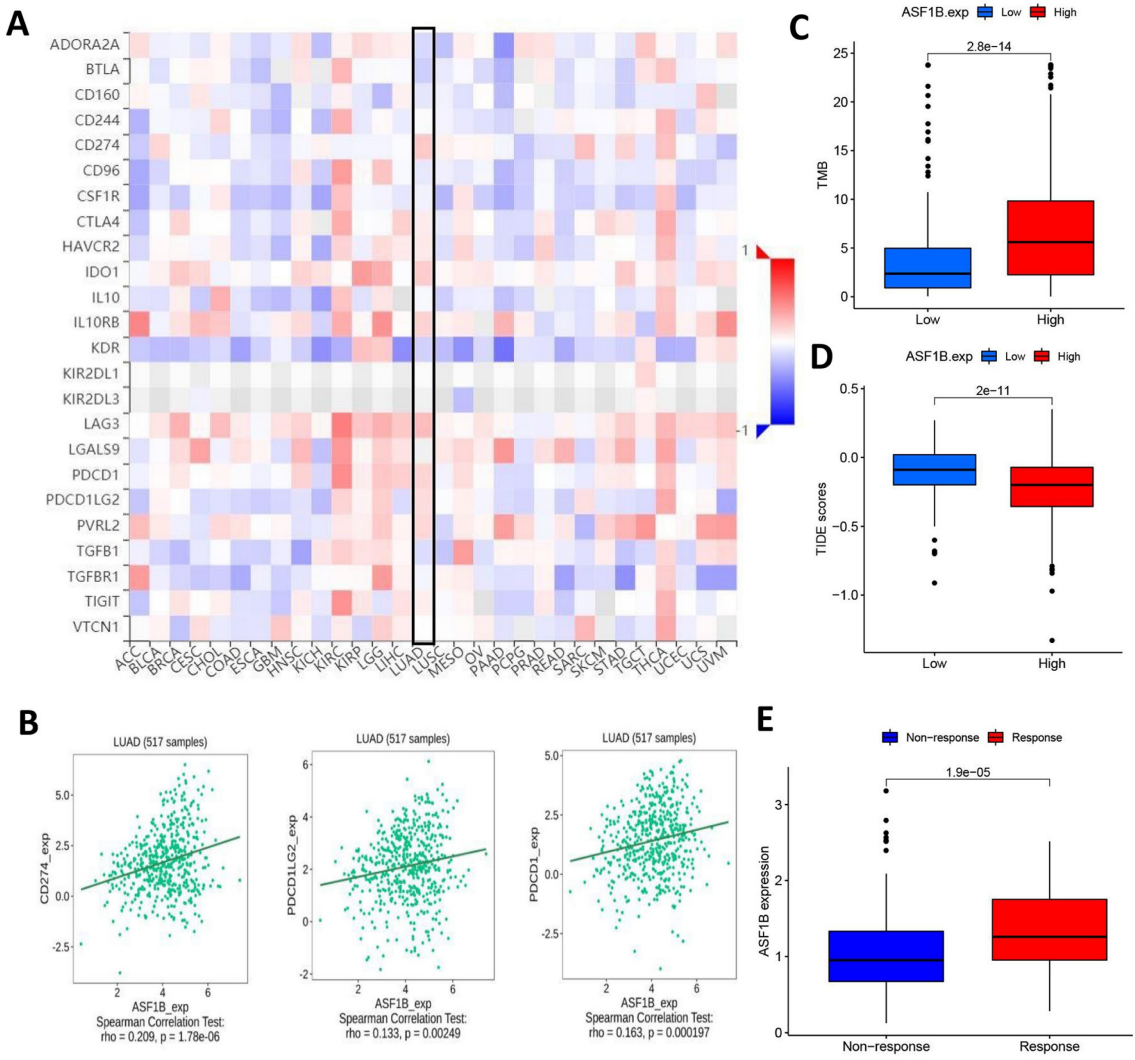
** ASF1B expression and immunotherapy effect. (A)** The relationship between ASF1B and some common immune checkpoints in human pan-cancer examined by Spearman correlation analysis. **(B)** The relationship between ASF1B and PD-L1, PD-1, PD-L2 in LUAD examined by Spearman correlation analysis. **(C)** The boxplot showing the relationship between ASF1B and TMB. **(D)** The boxplot showing the relationship between ASF1B and TIDE score.** (E)** Comparison of ASF1B expression levels between responser and non-responser of immunotherapy in the IMvigor210 cohort.

Given the above potential association of ASF1B with immunity, we were interested in further exploring the potential link between ASF1B and the efficacy of cancer immunotherapy. First, we analyzed the correlation of common immune checkpoints with ASF1B expression in human pan-cancers, and this result showed significant positive relationships between ASF1B expression and these immune checkpoints in multiple cancers, such as KIRP, LIHC, THCA, as shown in **Figure 8A**.

In LUAD, we also observed the findings. **Figure 8B** showed the correlation of ASF1B with PD-L1, PD-1, and PD-L2. Subsequently, we also compared the tumor mutation burden (TMB) in the different ASF1B level groups. As shown in **Figure 8C**, the high ASF1B group demonstrated a higher TMB. Furthermore, we also used the TIDE score to further explore the relationship between ASF1B and cancer immunotherapy response. As expected, the high ASF1B group presented a lower TIDE score than that of the low ASF1B group (**Figure 8D**). These data consistently suggested that patients with higher ASF1B expression may have a better effect on immunotherapy. To further confirm this inference, we also performed further exploration in an immunotherapy cohort (IMvigor210) with a relatively large sample size. We found that responders to immunotherapy had higher ASF1B expression, as shown in **Figure 8E**. This further suggested that the patients with high ASF1B expression might have more sensitivity to immunotherapy.

## Discussion

In the current study, we found that ASF1B expression was dysregulated (high expression state) in various human tumors. This finding was in line with previous research 22, 23. Surprisingly, the high expression state did not correspond to their poor prognosis. For example, in CESC, LUSC, as well as STAD, ASF1B was markedly overexpressed in cancer tissues, while this overexpressed state predicted better overall survival. Existing studies have given no definite explanation, and whether this is related to the different roles of ASF1B in different cancer contexts and different stages of cancer development needs to be further studied. The study of Liu et al 24 showed that ASF1B silencing inhibited the growth of cervical cancer cells *in vivo* and *in vitro*, induced cell cycle arrest and promoted apoptosis, while ASF1B overexpression promoted the proliferation of cancer cells. This confirmed that ASF1B plays a role as an oncogene in cervical cancer cells. Similar to the study, we also found that ASF1B functions as an oncogene in LUAD. We observed high expression state of ASF1B in LUAD tumor tissues in multiple datasets (TCGA, GSE10072 and GSE32863), and this high expression state had a negative impact on patient outcome (TCGA, GSE30219, GSE31210, GSE42127, GSE68465 and GSE72094). Additionally, we found that ASF1B silencing inhibited the proliferation, migration and invasion of LUAD cells *in vitro*. On the contrary, the overexpression of ASF1B showed the opposite biological behavior in A549 cells. We speculate that this promoting effect may be related to AKT phosphorylation. AKT, a serine/threonine protein kinase, is an important downstream effector of PI3K signaling, and elevated expression of activated AKT (p-AKT) has been confirmed to be present in many human cancers, such as breast cancer 25, esophageal cancer 26, pancreatic cancer 27, lung cancer 28, etc. AKT as the core to the PI3K/AKT signaling pathway, can phosphorylate some key premalignant factors to promote cell viability and inhibit apoptosis. Han et al 29 also found that silencing ASF1B suppressed the proliferation of prostate cancer cells and promoted their apoptosis and cell cycle arrest, and this inhibitory effect might be related to the inactivation of the PI3K/Akt pathway. This was similar to the results of our study. Our study found that overexpression of ASF1B significantly increased the level of p-AKT, while the inhibition of ASF1B expression had the opposite effect. The PI3K/AKT signaling pathway has been reported to be involved in the regulation of NSCLC proliferation, migration, and epithelial mesenchymal transition (EMT) 30. Our study also found that the inhibitor of AKT significantly inhibited the malignant behavior of lung cancer cells, and this inhibitory effect was greatly reduced in ASF1B-overexpressed cells. These data suggested the important role for ASF1B in tumor development. Similar findings can also be seen in the study of Zhang and Wang et al 31. Our findings once again emphasized that high ASF1B expression predicted a poor prognosis in patients with LUAD, suggesting that ASF1B might also serve as a prognostic marker gene in LUAD.

To further understand the role of ASF1B in LUAD, we further explored the immune-related mechanisms of ASF1B using bioinformatics methods. We found that there were significant correlations between ASF1B and multiple immune cells, as well as immune function or pathways. For example, ASF1B was significantly and positively associated with tumor killer cells (e.g., CD8+ T cells and NK cells) and APC co-inhibition, and Inflammation promoting. In hepatocellular carcinoma 32, 33, ASF1B was also found to be associated with multiple immune-infiltrating cells and immune function signaling pathways, but it was different from our study. They found that ASF1B transcription level was positively linked to the abundance of immune cell infiltration, including CD4+ T cells, DCs, Neutrophils, CD8+ T cells, and B cells. While our study observed that the seven had higher infiltration abundance in the low ASF1B group, including B cells, DCs, iDCs, Mast cells, Neutrophils, TIL, T helper cells. While CD8+ T cells, NK cells, and Th1 cells had higher levels of infiltration in the high ASF1B group. This might indicate that ASF1B had different immune-related roles in different cancer contexts. Given the significant impact of immunotherapy as an emerging treatment in improving the prognosis of cancer patients, we subsequently also investigated the potential association of ASF1B with immunotherapy. Previous studies 19, 34, 35 have revealed the immune checkpoint level, TMB, as well as TIDE in representing the efficacy of immunotherapy. As expected, there was positive correlation between ASF1B expression and immune checkpoint level such as TNRSF9, CD274, PDCD1LG2, LAG3 and PDCD1.

Patients with high ASF1B transcriptional level presented higher TMB as well as lower TIDE score, which already preliminarily suggested that ASF1B might be a potential biomarker to reflect the efficacy of immunotherapy. This inference was reconfirmed in another immunotherapy cohort (IMvigor210). This was also consistent with the findings of the study by Zhang et al. 32. Interestingly, ASF1B was considered to be associated with immunotherapy efficacy in both hepatocellular carcinoma as well as LUAD, however, the correlation between ASF1B expression and the abundance of immune cell infiltration was clearly different in these two cancers. This further highlighted the heterogeneity and complexity of the tumor microenvironment.

Overall, our study revealed that ASF1B was overexpressed in LUAD tumor tissues, and was significantly associated with the progression, prognosis, and immunotherapy effect for LUAD. ASF1B might be able to serve as a novel molecular marker to predict patient prognosis and a novel target for tumor therapy. It should be noted that although our data re-emphasize the importance of ASF1B in LUAD, the immune-related mechanisms of ASF1B in LUAD remain unclear, which still need to be intensively investigated in the future.

## Supplementary Material

Supplementary figures.

## Figures and Tables

**Table 1:** Basic information of the included patients with lung adenocarcinoma from TCGA.

Characteristics	Low expression of ASF1B	High expression of ASF1B	P value
**Age, n (%)**			0.035
<= 65	117 (22.5%)	140 (26.9%)	
> 65	144 (27.7%)	119 (22.9%)	
**Gender, n (%)**			0.179
Female	152 (28.2%)	137 (25.4%)	
Male	117 (21.7%)	133 (24.7%)	
**Smoker, n (%)**			0.779
No	37 (7%)	40 (7.6%)	
Yes	223 (42.5%)	225 (42.9%)	
**Pathologic T stage, n (%)**			0.031
T1	102 (19%)	74 (13.8%)	
T2	134 (25%)	158 (29.5%)	
T3&T4	31 (5.8%)	37 (6.9%)	
**Pathologic N stage, n (%)**			0.004
N0	189 (36.1%)	161 (30.8%)	
N1	39 (7.5%)	58 (11.1%)	
N2&N3	28 (5.4%)	48 (9.2%)	
**Pathologic M stage, n (%)**			0.079
M0	183 (46.9%)	182 (46.7%)	
M1	8 (2.1%)	17 (4.4%)	
**Pathologic stage, n (%)**			0.001
Stage I	169 (31.8%)	127 (23.9%)	
Stage II	53 (10%)	72 (13.6%)	
Stage III	32 (6%)	52 (9.8%)	
Stage IV	9 (1.7%)	17 (3.2%)	

## Abbreviations

ASF1B: histone H3-H4 chaperone anti-silencing function 1B
LUAD: lung adenocarcinoma
TCGA: the Cancer Genome Atlas
GEO: Gene Expression Omnibus
ssGSEA: Single-sample gene set enrichment analysis
TMB: tumor mutation burden
TIDE: tumour immune dysfunction and exclusion
qRT-PCR: Quantitative real-time polymerase chain reaction
