# Supplementary material for: Carcinogenic Role and Clinical Significance of Histone H3-H4 Chaperone Anti-silencing Function 1 B (ASF1B) in Lung Adenocarcinoma
Source: J Cancer. 2024 Jan 1;15(1):218–31. doi: 10.7150/jca.88777 (PMC10751675; doi:10.7150/jca.88777)
Supplement: Supplementary file 1 — Supplementary figures. [file jcav15p0218s1.pdf]

Carcinogenic Role and Clinical Significance of Histone H3-H4 Chaperone Anti-silencing Function 1 B (ASF1B) in Lung Adenocarcinoma

Congkuan Song [1 #], Yaolin Song[2 #], Xiaoxia Wan[2 #], Zhihong Zhao[2], Qing Geng [1]

[1] Department of Thoracic Surgery, Renmin Hospital of Wuhan University, Wuhan, China.
[2] Department of Thoracic Surgery, Ezhou Central Hospital, Ezhou, China.

[#] These authors contributed equally to this work.

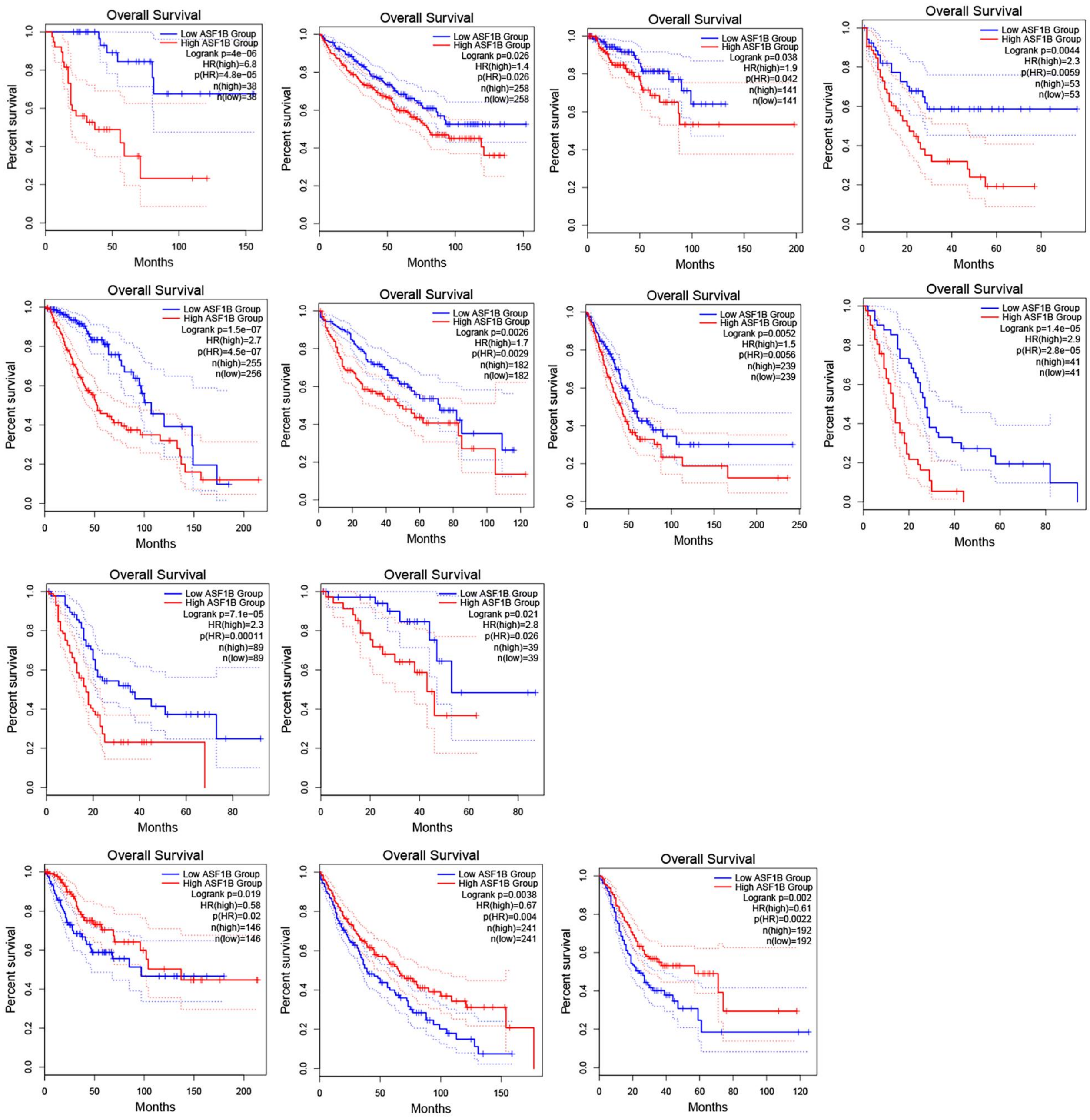

Figure S1: The relationship between the expression of ASF1B and the patient outcome from GEPIA2.

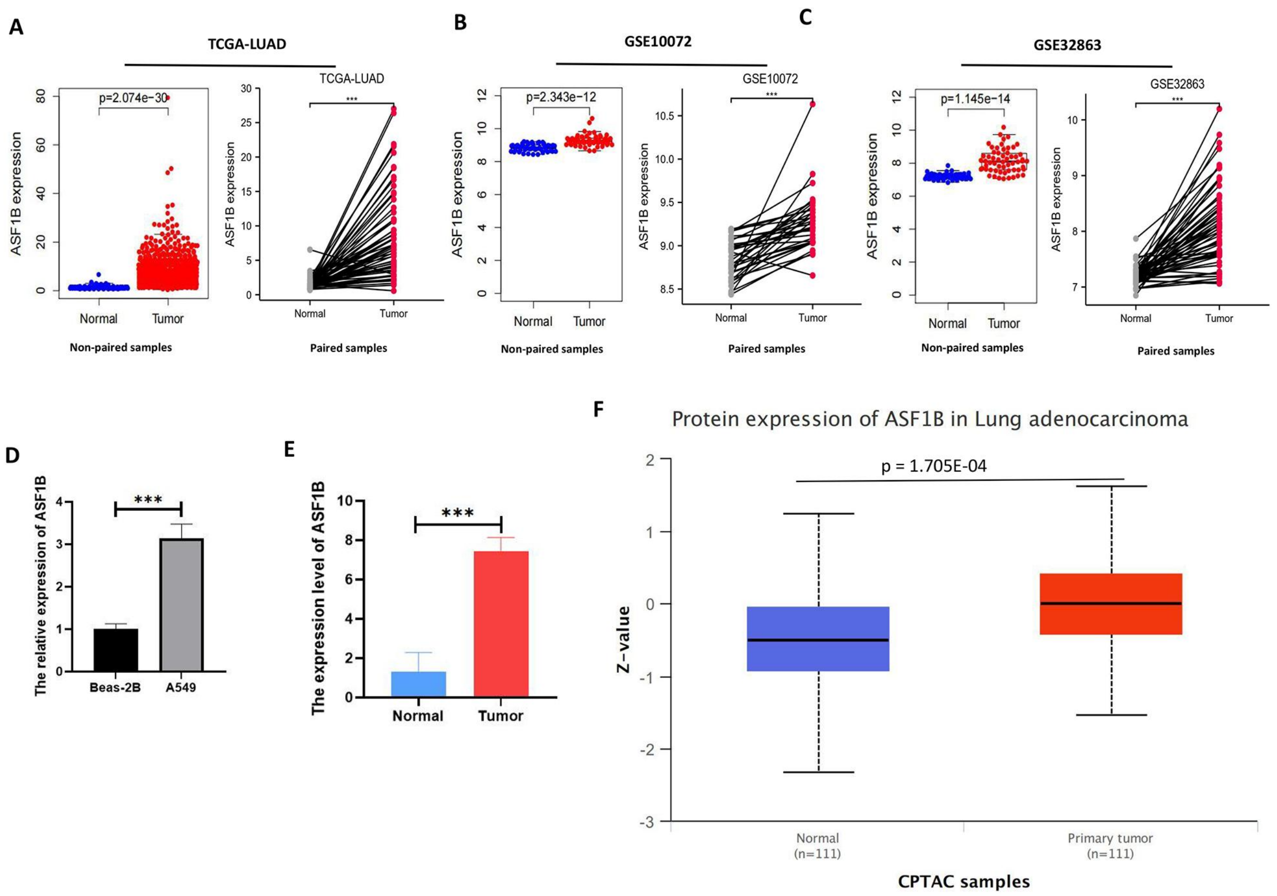

Figure S2: Comparison of ASF1B expression levels between LUAD tumor and normal tissues. (A-C) Comparison of transcription levels between LUAD tumor and normal tissues, including paired and unpaired samples, in the TCGA **(A),** GSE10072 **(B)**, and GSE32863 **(C)** cohorts**. (D)** The mRNA expression of ASF1B in the A549 and Beas-2B cells. **(E)** qPCR analysis revealed the mRNA expression of ASF1B in the LUAD tumor and normal tissues. **(F)** UALCAN portal analysis revealed the protein levels of ASF1B in tumor versus normal tissues.

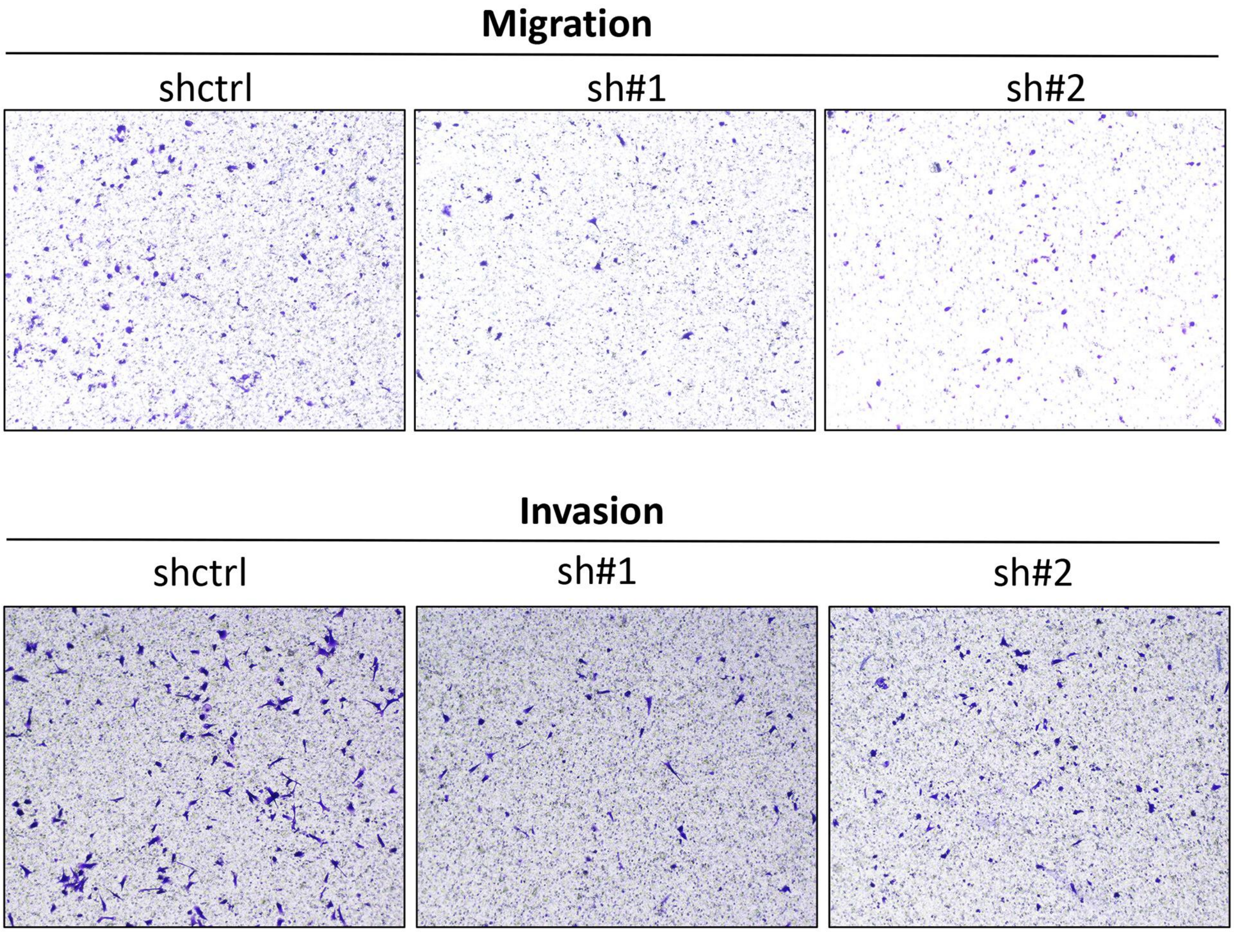

Figure S3: (A) Transwell assay revealed the migratory abilities of A549 cells. (B) Transwell assay revealed the invasive abilities of A549 cells.
